# Parents of healthy children assign lower quality of life measure to scenarios labeled as cancer than to identical scenarios not labeled as cancer

**DOI:** 10.1186/s40359-019-0280-5

**Published:** 2019-02-21

**Authors:** Brenna M. McElderry, Emily L. Mueller, Abigail Garcia, Aaron E. Carroll, William E. Bennett Jr

**Affiliations:** 1 Indiana University School of Medicine, Indianapolis, USA; 2 Center for Pediatric and Adolescent Comparative Effectiveness Research, Department of Pediatrics, Indiana University School of Medicine, Indianapolis, USA; 3 Section of Pediatric Hematology and Oncology, Department of Pediatrics, Indiana University School of Medicine, Indianapolis, USA; 4 Indiana University, Bloomington, USA; 5 Section of Pediatric Gastroenterology, Hepatology, and Nutrition, Department of Pediatrics, Indiana University School of Medicine, Indianapolis, USA

**Keywords:** Cancer, Childhood, Health utility, Quality of life, Decision making, Bias

## Abstract

**Background:**

While it is commonly understood that a cancer diagnosis evokes feelings of fear, the effect of labeling a child’s illness as “cancer” remains unstudied. We hypothesized that lower health utility scores would be assigned to disease states labeled as cancer compared to identical disease states without the mention of cancer.

**Methods:**

In this randomized study, caregivers of healthy children were asked to assign health utility values to different scenarios written as improving, stable, or worsening. Participants from general pediatric clinics at Eskenazi Health were randomly assigned to either the scenarios labeled as “cancer” or “a serious illness”. Participants then rated the scenarios using the Standard Gamble, with laddering of health utilities between 0 (a painless death) and 1 (perfect health). We also gathered subject demographics and assessed the subject’s numeracy.

**Results:**

We approached 319 subjects and 167 completed the study. Overall median health utilities of “cancer” scenarios were lower than “serious illness” scenarios (0.61 vs. 0.72, *p* = 0.018). Multivariate regression (with an outcome of having a utility above the 75th percentile) showed no significant effects by race, ethnicity, numeracy, or income level. “Cancer” scenarios remained significantly lower after adjustment for confounders using logistic regression, but only for the more serious scenarios (OR 0.92, *p* = 0.048).

**Conclusions:**

On average, caregivers with healthy children were shown to take more risk with their treatment options and view their child as having a worse quality of life when they knew the disease was cancer. Awareness of this bias is important when discussing treatments with families, particularly when a risk of cancer is present.

## Background

Cancer is a rare diagnosis among children ages 0–19 years and the most common types are associated with high survival rates overall [1, 2]. However, there is evidence that childhood cancer is commonly misunderstood by the general public [3, 4]. While the literature is lacking in direct survey of public opinion, studies analyzing media portrayal of childhood cancer show a particularly negative connotation of the cancer label. Media has been shown to heavily influence public opinion on a wide range of topics [5]. One such study pursued how childhood cancer is portrayed in recent films and found a cinematic mortality rate of 66%, compared to the actual mortality rate of 16% for all childhood cancers [3]. Another study analyzed all magazine articles published on cancer between 1970 and 2001 [4]. One of the study’s major findings was a common narrative structure drastically contrasting the before and after of a childhood cancer diagnosis, which they hypothesized to exacerbate societal fear and stigma surrounding childhood cancer, despite most children returning to everyday life [4].

These misguided perceptions of childhood cancer could impact medical decision making by caregivers of children, including when the risk of cancer is present. For example, those treating rheumatologic conditions with tumor necrosis factor-alpha inhibitors increase their risk of non-Hodgkin’s lymphoma [6]. Azathioprine therapy for those with inflammatory bowel disease has been associated with an increased risk of overall cancer, and the use of CT scans on children carries an established increased risk of leukemia and brain cancer [7, 8]. Caregivers are often faced with treatment decisions requiring an accurate understanding of childhood cancer. This warrants a need to properly assess the public’s opinions on the quality of life of that particular disease state.

Health utility measurement is an ideal method to assess the impact of the term “cancer” on perceived quality of life. Health utilities measure the quality of a specific health state based on health decision making [9]. It is well studied that the more risk someone is willing to take with a treatment to cure a disease, the worse they perceive that disease state [9]. Health utilities are generated by presenting a participant with a particular health state and description. The participant is then asked to imagine being presented with a new drug that cures the presented health state, but carries a level of risk of a defined worsening of their quality of life. The percentage of risk is adjusted to a point of indifference, meaning we find the highest percentage of risk the person is willing to take for a curative measure. This percentage is converted into a health utility score for a disease that ranges from 0 to 1, with 0 equivalent to a quick and painless death, and 1 equivalent to perfect health [10–12]. These scores can then be used to compare quality of life between different health states and health outcomes. This approach was used to evaluate the perceived impact of varying stages of breast cancer, which was modeled by attaining a subject’s opinion on multiple health states and toxicities to treatment [13]. No prior studies have taken a similar approach to investigate perceived quality of life in childhood illness by caregivers of healthy children, particularly investigating the impact of the term “cancer” in scenario descriptions.

We chose to investigate the social construct surrounding childhood cancer. The goal of this study was to determine if use of the term “cancer” affects a caregiver’s assignment of health utilities for their child. Our study assessed the reaction of caregivers of healthy children to the disease states of childhood cancer versus an equally serious illness but without the label of “cancer.” We hypothesized that caregivers would assign a lower health utility to disease states described as cancer than disease states described as a serious illness despite the same description of disease state. This would mean the use of the word “cancer” made scenarios appear to have a comparatively worse quality of life. The results of our study may improve provider understanding of the general public’s preconceived notions of childhood cancer and identify gaps in patient education.

## Methods

### Study setting and subject characteristics

Subjects were enrolled at general pediatrics clinics in Eskenazi Health, located in an urban area of Indianapolis, Indiana. The Eskenazi system provides healthcare for over 1 million outpatient visits by the diverse, urban residents of Marion County [14]. We approached adults waiting for pediatric visits if they had a child who was less than 18 years of age. We excluded subjects who had ever had a child with cancer. We approached patients that spoke either English or Spanish, as we have bilingual research assistants available.

### Health utility standard gamble

Health utilities are commonly studied using the Standard Gamble (SG) technique, which measures individual preferences for different therapeutic options amidst uncertain results [9, 10]. We randomized subjects to receive either scenarios which described “a serious illness”, or scenarios explained as “cancer”, differing only by that label. The two groups of scenarios were otherwise identical, and subjects were presented with three different clinical situations: one depicting a disease that is responding to treatment (Scenario 1), one depicting a disease that is stable on treatment (Scenario 2), and one depicting a disease that is not responding to treatment (Scenario 3). The text of these scenarios can be found in Appendix A. The scenarios were prearranged in order of severity along with our anchor scenarios merely stating “a quick and painless death” as first and “perfect health” as last. Thus, a list of 5 scenarios in total were presented to the participant to read all together from worst case scenario to best case scenario (death, scenario 3, scenario 2, scenario 1, perfect health) because the order felt to be universally agreed upon. A quick and painless death was used as the anchor point for simplicity and precedence [11]. Many different “0” anchor points are possible, but our past experience with this methodology indicates that a simple presentation of the “death” end of the spectrum produces more consistent results and allows easier comparison to previous studies [11, 12].

We then performed the Standard Gamble technique to ascertain health utilities [15]. Beginning with death and the scenario where disease was not responding to treatment (Scenario 3), we asked the subject to imagine that their child could either continue with the scenario in question, or take a medication which cures him or her, but carries a risk of death. We started with the medication having a 50% chance of curing the disease and 50% chance of causing a quick and painless death. We iteratively moved the likelihood of death up or down depending on their response until the subject was indifferent about the outcome. In other words, we sought out the highest amount of risk a caregiver was willing to take with a curative medication. Once this point of indifference was ascertained, we changed the gamble so that the most recently assessed scenario of a disease not responding to treatment (Scenario 3) was moved in place of death, and the next scenario up the chain, one depicting a disease stable on treatment (Scenario 2), was assessed. We determined how much risk of the disease becoming unresponsive to treatment a caregiver was willing to take for a cure within the new disease state (Scenario 2). This laddering was then done a third time, assessing a disease responding to treatment (Scenario 1) by giving the caregiver the option to stay in the current state or take a curative medication that had a risk of the child’s illness becoming merely stable on treatment (Scenario 2). A gamble percentage was ultimately established between each scenario. These percentages were then used to compute the health utility for each scenario with the formulas found in Appendix B.

### Numeracy assessment

After the gamble was complete, we asked each caregiver a series of questions of increasing difficulty to assess numeracy. Numeracy is the subject’s understanding of percentage values and probabilities and how to interpret them and is also known as mathematical literacy. The assessment can be found in Appendix C.

### Demographics

We gathered demographic data for both the participant and the child (age, race, ethnicity, and gender), household income, highest level of caregiver education, number of children in the family, and whether the family was a single parent household.

### Statistical analysis

Prior to the start of the study, we performed power calculations for the comparison between the set of scenarios explained as cancer and the set of scenarios explained as a serious illness. We wished to detect a difference of 0.05 between the median “serious illness” utility and the median “cancer” utility for children, with an estimated initial utility of 0.85 for a serious illness. Since no studies have analyzed these health states from the general public’s point of view, this starting point was based off of childhood cancer studies assessing current patient’s quality of life [16, 17]. With a power of 80% and an ⍺ of 0.05, we estimated that we needed 126 subjects total.

The health utility scores generated for each scenario were calculated based off of the formulas found in Appendix B. We used univariate statistics to compare demographic data of each arm (“cancer” or “serious illness”) using the Student’s t-test for continuous data and the chi-square test for categorical data. We then compared the median health utilities of each scenario and all scenarios in aggregate using the Mann-Whitney test for medians. We chose a non-parametric test to compare the two arms, since health utilities are unlikely to be normally distributed. Finally, we performed multivariate logistic regression using health utility greater than the 75th percentile as the dependent variable, and numeracy, income, employment, race, and ethnicity (of subject) as independent variables. Since the distribution was nonparametric, we chose logistic regression over linear regression. All analyses were considered significant at *p* < 0.05. Models were built and statistics performed using R, version 3.22 (http://www.r-project.org). The Institutional Review Board at Indiana University School of Medicine approved the study with expedited status.

## Results

### Subject Participation

**Fig. 1:**
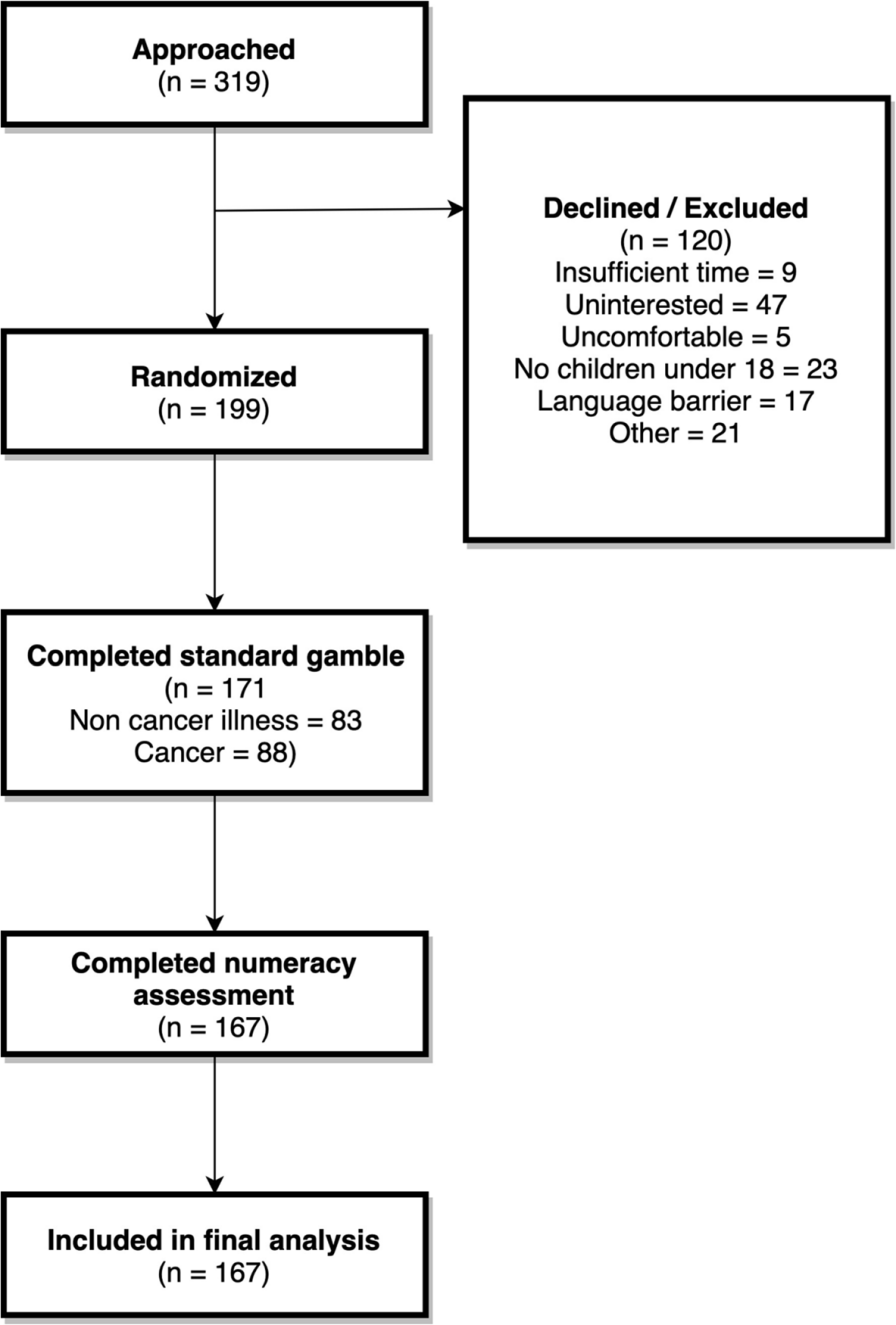
CONSORT Diagram. Flowchart of the number of subjects enrolled at each point in the study. “Other” includes those who did not understand the questions, determined by the administering researcher or subject themselves, or had specific reasons for not participating, such as a need to watch their kids closely. Most who agreed to participate but were unable to complete the survey ran out of time before being called back for their doctor’s appointment

A total of 319 people were approached to participate in the survey. Of those, 199 subjects were consented, and 167 subjects completed the study (see Fig. 1). Of those that completed the study, 81 subjects completed the “serious illness” scenarios and 86 subjects completed the “cancer” scenarios.

### Participant Demographics

Participant demographic characteristics are shown in Table 1. By univariate analysis, there were no significant differences in the number of participants randomly assigned to the “cancer” scenarios and the “serious illness” scenarios within each assessed demographic. Approximately half of the caregivers and their children were black and 18% were Hispanic. A little under half were unemployed at the time of enrollment and over half had an annual gross family income below $25,000. Half of the participants accurately answered the first numeracy assessment question, roughly a third answered the second question correctly, and only 4% answered the third numeracy question correctly.

**Table 1:** Comparison of Collected Demographic Data between Participants of Cancer and Non-Cancer Scenarios

		All Scenarios *N* = 167	“Cancer” Scenario *N* = 86	Non-cancer Serious Illness Scenario *N* = 81	*p*-value
Caregiver Age	median (interquartile range)	32 (19,45)	31 (16,46)	33 (10)	0.70
	mean (standard deviation)	32.9 (10)	32.9 (11)	32.8 (9)	0.94
Patient Age	median (interquartile range)	7 (0,17)	8 (0,18)	7 (0,16)	0.85
	mean (standard deviation)	7.7 (6)	7.8 (6)	7.5 (5)	0.72
Caregiver Gender	Female	89/167 (53%)	43/86 (50%)	46/81 (57%)	0.38
Caregiver Race	Black	85/167 (51%)	46/86 (54%)	39/81 (48%)	0.50
	White	53/167 (32%)	23/86 (27%)	30/81 (37%)	
	Asian	4/167 (2%)	2/86 (2%)	2/81 (3%)	
	Other	25/167 (15%)	15/86 (17%)	10/81 (12%)	
Patient Race	Black	80/167 (48%)	44/86 (51%)	36/81 (44%)	0.26
	White	45/167 (27%)	18/86 (21%)	27/81 (33%)	
	Asian	3/167 (2%)	1/86 (1%)	2/81 (3%)	
	Other	39/167 (23%)	23/86 (27%)	16/81 (20%)	
Caregiver Ethnicity	Hispanic	31/167 (19%)	14/86 (16%)	17/81 (21%)	0.71
	Non-Hispanic	135/167 (81%)	72/86 (84%)	63/81 (78%)	
	Unknown	1/167 (1%)	0/86 (0%)	1/81 (1%)	
Patient Ethnicity	Hispanic	39/167 (23%)	18/86 (21%)	21/81 (26%)	0.56
	Non-Hispanic	126/167 (75%)	68/86 (79%)	58/81 (72%)	
	Unknown	2/167 (1%)	0/86 (0%)	2/81 (3%)	
# Children in Family	median (interquartile range)	2 (0,4)	2 (0,4)	2 (1,3)	0.93
Highest Level of Education Achieved	Some high school	18/167 (11%)	5/86 (6%)	13/81 (16%)	0.24
	High school graduate	67/167 (40%)	35/86 (41%)	32/81 (40%)	
	Some college	46/167 (28%)	24/86 (28%)	22/81 (27%)	
	College graduate	20/167 (12%)	13/86 (15%)	7/81 (9%)	
	Graduate school	14/167 (8%)	7/86 (8%)	7/81 (9%)	
Employed		91/167 (55%)	50/86 (58%)	41/81 (51%)	0.33
Annual Family Income (US dollars)	< 10 k	54/167 (32%)	29/86 (34%)	25/81 (31%)	0.41
	10-25 k	44/167 (26%)	26/86 (30%)	18/81 (22%)	
	25-50 k	35/167 (21%)	14/86 (16%)	21/81 (26%)	
	50-75 k	8/167 (5%)	3/86 (4%)	5/81 (6%)	
	75-100 k	8/167 (5%)	5/86 (6%)	3/81 (4%)	
	> 100 k	5/167 (3%)	5/86 (6%)	0/81 (0%)	
	Refused	13/167 (8%)	4/86 (5%)	9/81 (11%)	
Single Parent Household		79/167 (47%)	36/86 (42%)	43/81 (53%)	0.15
Numeracy	Question 1 correct	82/167 (49%)	42/86 (49%)	40/81 (49%)	0.94
	Question 2 correct	50/167 (30%)	28/86 (33%)	22/81 (27%)	0.45
	Question 3 correct	6/167 (4%)	3/86 (4%)	3/81 (4%)	0.94

### Health Utilities

**Fig. 2:**
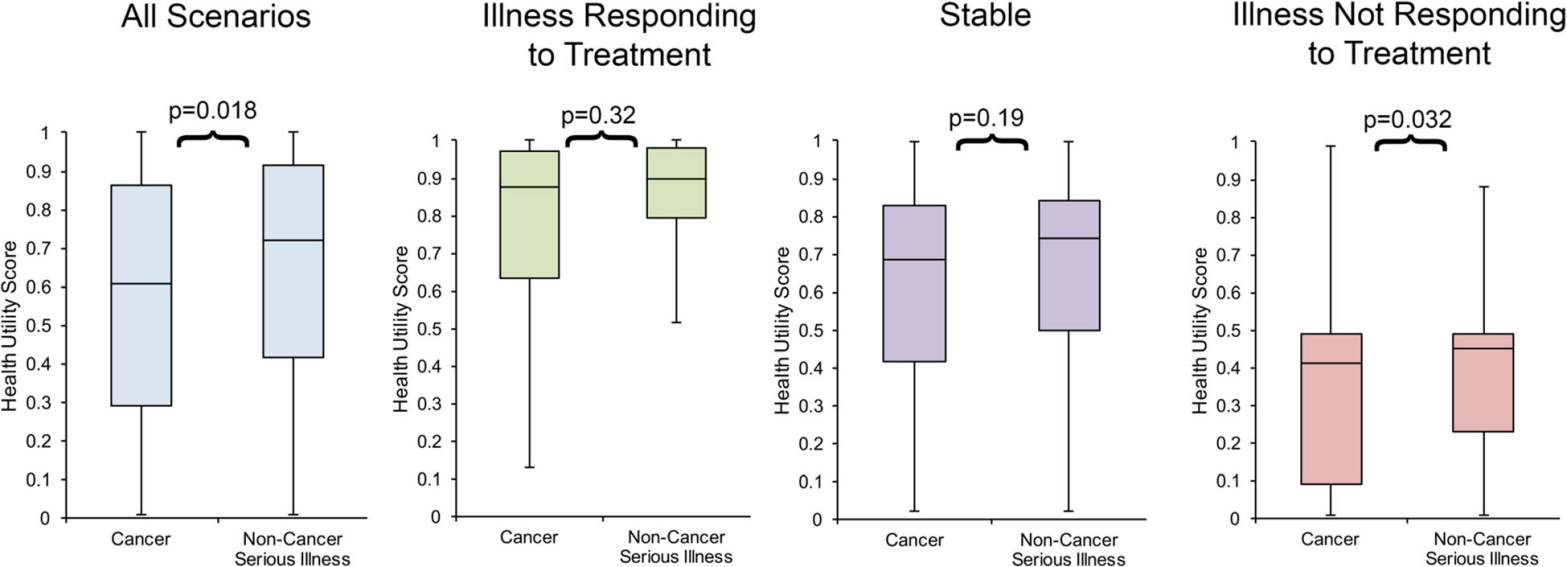
Median health utility scores assigned to cancer and non-cancer scenarios with interquartile range and *p*-values

We calculated the median and interquartile range (IQR) for the health utility in each individual scenario as well as the aggregate median and IQR for each arm, which can be seen in Fig. 2. The aggregate health utility for all three “cancer” scenarios was 0.61 (IQR: 0.29,0.86), which was significantly lower (Mann-Whitney u score: 27512, z-score: − 2.37, *p*-value: 0.018) than the aggregate “serious illness” scenarios’ median of 0.72 (IQR: 0.42,0.92). Median health utility values assigned for scenario 3 of “cancer” (0.39, IQR: 0.10,0.49) were also significantly lower (Mann-Whitney u score: 2810.5, z-score: − 2.15, p-value = 0.032) than equivalent “serious illness” scenarios (0.49, IQR: 0.23,0.61).

The health states assigned to scenario 1 (illness responding to treatment) and scenario 2 (stable) for “cancer” and “serious illness” were not significantly different. For the scenarios describing an illness responding to treatment (Scenario 1), those that mentioned cancer were assigned a median health utility of 0.88 (IQR: 0.63,0.97) and those that were described as a serious illness were assigned a median health utility of 0.90 (IQR: 0.79,0.98) with a *p*-value of 0.32 (Mann-Whitney u value: 3169, z-score: − 1.00). For the scenarios describing a stable illness (Scenario 2), those that mentioned cancer were assigned a median health utility of 0.69 (IQR: 0.41,0.83) and those that were mentioned as a serious illness were assigned a median health utility of 0.74 (IQR: 0.50,0.84) with a *p*-value of 0.19 (Mann-Whitney u value: 3073, z-score: − 1.3).

### Regression Model

The results of the logistic regression model are shown in Table 2. We controlled for family income, employment status, numeracy, race, and ethnicity, none of which were significant. In our model, the only statistically significant determinant of health utility score was our variable of interest: whether the term “cancer” was used or not in the scenarios, although the confidence interval was very close to 1.00, with a *p*-value of 0.048.

**Table 2:** Multivariate Regression Analysis of Demographic Information on Health Utility Assignment

		Non-Cancer Serious Illness Scenario			All Scenarios		
		Odds Ratio	95% Confidence Interval	*p*-value	Odds Ratio	95% Confidence Interval	*p*-value
“Cancer” Language Used		0.92	0.84,1.00	0.048	0.94	0.87, 1.02	0.13
Numeracy	Question 1 correct	1.04	0.96, 1.14	0.31	1.06	0.98, 1.15	0.16
	Question 2 correct	1.03	093, 1.13	0.60	1.03	0.94, 1.13	0.50
	Question 3 correct	1.06	0.83, 1.36	0.65	1.07	0.84, 1.35	0.59
Annual Family Income (US dollars)	< 10 k	Reference	–	–	–	–	–
	10-25 k	1.00	0.89, 1.12	0.97	1.04	0.93, 1.16	0.49
	25-50 k	0.96	0.84, 1.09	0.52	1.07	0.95, 1.21	0.26
	50-75 k	1.04	0.84, 1.28	0.71	1.05	0.87, 1.28	0.61
	75-100 k	1.01	0.80, 1.28	0.90	1.14	0.92, 1.42	0.24
	> 100 k	0.92	0.70, 1.21	0.54	1.05	0.81, 1.36	0.74
	Refused	1.24	1.04, 1.47	0.017	1.23	1.05, 1.45	0.01
Employed		0.93	0.84, 1.03	0.15	0.99	0.90, 1.08	0.77
Race	Black	Reference	–	–	–	–	–
	Asian	0.80	0.58, 1.11	0.18	0.90	0.66, 1.22	0.49
	White	0.98	0.87, 1.10	0.70	0.99	0.89, 1.11	0.93
	Other	0.90	0.79, 1.03	0.13	0.94	0.83, 1.07	0.38
Ethnicity	Hispanic	Reference	–	–	–	–	–
	Non-Hispanic	1.02	0.89, 1.17	0.76	1.07	0.94, 1.22	0.33
	Unknown	1.10	0.74, 1.65	0.64	1.14	0.78, 1.67	0.50

## Discussion

In this health utility study, we used the Standard Gamble method to show that using the term “cancer” when describing a serious illness in children leads to lower health utilities as expressed by caregivers of healthy children. “Cancer” scenarios were assigned a median health utility score of 0.61, compared with a significantly higher score of 0.72 for “serious illness” scenarios with the same description. This means that on average, the general public views their child as having a worse quality of life when they hear the disease is cancer rather than a generic serious illness, even if the disease experience is otherwise identical. This finding has important implications for discussing interventions with parents when their child has a risk of cancer. A number of immunosuppressants, radiological tests, and emerging therapies have a small risk of cancer; this study can provide a framework for further research on understanding the unique effect that the risk of developing cancer has on the therapeutic choices that caregivers make on behalf of their children, and tailor discussions to be sensitive to this fact [6–8]. By awarding “cancer” health states a lower quality of life measure than identical “serious illness” health states, parents reveal a possible gap in knowledge that could be filled in the discussion of treatments with a risk of cancer.

The strongest effect on perceived health utility seemed to occur for the third scenario, which was the disease state not responding to treatment. The “cancer” scenario had a median health utility score of 0.39, while the “serious illness” scenario not responding to treatment had a significantly higher median health utility score of 0.49. Thus, the mention of cancer to the participant was influential in the most critical disease state, further supporting our hypothesis. We speculate that preconceived notions about cancer, influenced by either media portrayal or experiences with people other than their children, play a role in caregiver decision making [3, 4]. Inherent biases may cause caregivers to rely less on the facts of their child’s state and more on a sociologically and/or personally constructed perception of cancer. This misperception may influence some caregivers to avoid or doubt important diagnostics or treatments with a risk of cancer for their child. Awareness of this bias is important for both providers and caregivers, who may be unaware of this potential barrier to care. We hope to begin the conversation on a possible area in patient-physician dialogue needing further explanation.

A search of the literature did not reveal any studies specifically asking parents of healthy children about health utilities of childhood cancer. Prior research on childhood cancer utilities has been accomplished by administering questionnaires to parents of children already affected by cancer and assigning a health utility score to their child’s experience during treatment. The literature shows higher health utilities in childhood acute lymphoblastic leukemia (ALL) (0.74–0.88 depending on stage of treatment), the most studied of the childhood cancers, than the childhood cancer health utility values we generated [16, 17]. We believe this is partly because childhood ALL typically has a good prognosis [16, 17]. Our scenarios covered good, fair, and poor prognoses. Another contributing factor could be from these caregivers having a more realistic expectation of the quality of life with childhood cancer. This may further illustrate the general public’s potentially misguided perception of childhood cancer as a worse quality of life than it is for common cancers prior studies investigated. When parents do have a child with cancer, they are more extensively informed about the prognosis and therefore seem to make more reasoned and balanced decisions. This suggests that “cancer” may have an emotive influence on parents of healthy children. We believe this has the potential to impact parental decision making in relation to their children undertaking tests or treatments that may carry with it a risk of cancer. Ultimately, our research is meant to start a conversation in a new avenue about the public perception of childhood health utility states.

While our investigation targeted a different audience (i.e. caregivers rather than patients), our health utility scores for childhood cancer align more closely to the work done from the societal perspective of adult metastatic breast cancer, where subjects assigned a health utility score of 0.79 for disease responding to treatment, 0.72 for stable disease on treatment, and 0.45 for worsening disease progression [13]. This reinforces the general public’s perception of cancer with similar health utility values to those we generated. Thus, our study fills an important gap in the literature by highlighting the perceptions of childhood cancer by caregivers of healthy children.

This study has important limitations. First, comparing something general like a “serious illness” to something more specific like cancer could raise concerns that any specific disease may be viewed more negatively than a generic serious illness. While this is possible, we explained both diagnoses with the same exact specific description. We covered functional state, symptoms, pain level, mental health, and parental concern to clarify and give specifics on the serious illness so that it was defined. This study is ultimately meant to be a starting point for future studies to then compare childhood cancer to other similarly serious diseases like inflammatory bowel disease, cystic fibrosis, diabetes, and so many more. Second, the study’s population primarily included high numbers of participants of low socioeconomic status, low levels of education, minority race populations, and low numeracy. The sample for this study is therefore not necessarily representative of the general public but can still provide insight into the preferences of many populations, specifically people of color and lower socioeconomic status, who are traditionally underrepresented in clinical research. Future studies should seek to determine perspectives about cancer from caregivers of healthy children in a larger variety of scenarios and differing demographic categories.

## Conclusion

The use of the term “cancer” lowers perceived health utilities in caregivers of healthy children when compared to an identical serious illness. We aim to establish a concern with the public’s understanding of this serious disease and question how it impacts decision making when a risk of cancer is present. Awareness of this bias is important when discussing treatment options with a risk of cancer with families. Our study provides a framework for future studies to clarify this notion and contributes to the understanding of the public’s perception of childhood cancer disease states.

## Appendix 1

### Serious Illness Scenarios

#### Scenario 1

Your child has a serious illness that is responding to treatment. Your child needs to be brought into the outpatient clinic for continuous cycles of treatment. The treatment makes your child anxious and he/she does not like being in the hospital, but your child does not seem to be too concerned about their illness. He/she is occasionally nauseous, but your child’s appetite is good. Their energy level seems to be the same as other kids his/her age. Your child experiences pain infrequently that can be treated with oral medication. You worry for your child, but you are hopeful they will be healthy in the future.

#### Scenario 2

Your child has a serious illness that is stable on treatment. Your child needs to be brought into the outpatient clinic for continuous cycles of treatment. The treatment makes your child anxious and he/she does not like being in the hospital. Your child does not have a good appetite and motivating him/her to eat is difficult. Your child is constantly nauseous. Your child gets tired often, but can still interact with you and others for short periods of time. This makes your child aware that they are not like other kids their age. Your child sometimes experiences pain which can be treated with oral medication. There is a worry that the illness will get worse in the future.

#### Scenario 3

Your child has a serious illness that is not responding well to treatment. Your child is on his/her second line of treatment, as the first treatment was unsuccessful at stopping the progression of the illness. Your child is getting worse even on the second line of treatment. The treatment makes your child anxious and/or depressed and he/she does not like being in the hospital. The depressed mood seems to be constant. Your child experiences severe fatigue and is losing a lot of weight. Your child is on a stronger oral pain medication and is regularly nauseous and vomiting. Your child is not able to play with other kids and is aware he/she is not like the other kids. You and your child are worried they will die of their illness.

### Cancer Scenarios

#### Scenario 1

Your child has cancer that is responding to treatment. Your child needs to be brought into the outpatient clinic for continuous cycles of treatment. The treatment makes your child anxious and he/she does not like being in the hospital, but your child does not seem to be too concerned about their cancer. He/she is occasionally nauseous, but your child’s appetite is good. Their energy level seems to be the same as other kids his/her age. Your child experiences pain infrequently that can be treated with oral medication. You worry for your child, but you are hopeful they will be healthy in the future.

#### Scenario 2

Your child has cancer that is stable on treatment. Your child needs to be brought into the outpatient clinic for continuous cycles of treatment. The treatment makes your child anxious and he/she does not like being in the hospital. Your child does not have a good appetite and motivating him/her to eat is difficult. Your child is constantly nauseous. Your child gets tired often but can still interact with you and others for short periods of time. This makes your child aware that they are not like other kids their age. Your child sometimes experiences pain which can be treated with oral medication. There is a worry that the cancer will progress in the future.

#### Scenario 3

Your child has cancer that is not responding well to treatment. Your child is on his/her second line of treatment, as the first treatment was unsuccessful at stopping the progression of the disease. Your child is getting worse on the second line of treatment. The treatment makes your child anxious and/or depressed and he/she does not like being in the hospital. The depressed mood seems to be constant. Your child experiences severe fatigue and is losing a lot of weight. Your child is on a stronger oral pain medication and is regularly nauseous and vomiting. Your child is not able to play with other kids and is aware he/she is not like the other kids. You and your child are worried they will die of their cancer.

## Appendix B

### Health Utility Score Formulas

To calculate the utility of each scenario for each subject, we converted each final % chance given by the subject into a utility value using the following formula, assuming the ranking of Perfect health ≥ Scenario 1 (responding to treatment) ≥ Scenario 2 (stable on treatment) ≥ Scenario 3 (not responding to treatment) ≥ Death:

Gamble (G)= the % chance of death given by the subject in return for curing the condition.

Scenario 3 Utility = U_Sc3_ = G_Sc3._

Scenario 2 Utility =U_Sc2_ = G_Sc2_ ∗ (1 − U_Sc3_)

Scenario 1 Utility= U_Sc1_ = G_Sc1_ ∗ (1 − U_Sc2_)

## Appendix C

### Numeracy Assessment

Each answer was coded as correct or incorrect:

1. Imagine that we flip a fair coin 1000 times. What is your best guess about how many times the coin would come up heads in 1000 flips? (Correct answer: 500)
2. Imagine that you are playing the BIG BUCKS LOTTERY, and the chance of winning a $10 prize is 1%. What is your best guess about how many people would win a $10 prize if 1000 people each buy a single ticket to the BIG BUCKS LOTTERY? (Correct answer: 10)
3. Imagine you have entered the PUBLISHING SWEEPSTAKES, where the chance of winning a car is 1 in 1000. What percent of tickets to the PUBLISHING SWEEPSTAKES win a car? (Correct answer: 0.10%)

## Abbreviations

ALL: Acute lymphoblastic leukemia
CI: Confidence Interval
IQR: Interquartile Range
OR: Odds Ratio
QALYs: Quality Adjusted Life Years
QOL: Quality of Life
SG: Standard Gamble technique
vNM: von Neumann-Morgenstern utility function
